# Tonal Symmetry Induces Fluency and Sense of Well-Formedness

**DOI:** 10.3389/fpsyg.2018.00165

**Published:** 2018-02-19

**Authors:** Fuqiang Qiao, Fenfen Sun, Fengying Li, Xiaoli Ling, Li Zheng, Lin Li, Xiuyan Guo, Zoltan Dienes

**Affiliations:** ^1^School of Education and Psychology, University of Jinan, Jinan, China; ^2^School of Psychology and Cognitive Science, East China Normal University, Shanghai, China; ^3^Department of Psychology, Zhejiang Normal University, Jinhua, China; ^4^School of Psychology, Shandong Normal University, Jinan, China; ^5^Shanghai Key Laboratory of Magnetic Resonance, East China Normal University, Shanghai, China; ^6^Key Laboratory of Brain Functional Genomics, Ministry of Education, Shanghai Key Laboratory of Brain Functional Genomics, East China Normal University, Shanghai, China; ^7^National Demonstration Center for Experimental Psychology Education, East China Normal University, Shanghai, China; ^8^School of Psychology, Sackler Centre for Consciousness Science, University of Sussex, Brighton, United Kingdom

**Keywords:** tonal symmetry, fluency, AGL, grammaticality judgments, implicit learning

## Abstract

Fluency influences grammaticality judgments of visually presented strings in artificial grammar learning (AGL). Of many potential sources that engender fluency, symmetry is considered to be an important factor. However, symmetry may function differently for visual and auditory stimuli, which present computationally different problems. Thus, the current study aimed to examine whether objectively manipulating fluency by speeding up perception (i.e., manipulating the inter-stimulus interval, ISI, between each syllable of a string) influenced judgments of tonal strings; and thus how symmetry-based fluency might influence judgments. In Experiment 1, with only a test phase, participants were required to give their preference ratings of tonal strings as a measurement of fluency. In experiment 2, participants were instructed to make grammaticality judgments after being incidentally trained on tonal symmetry. Results of Experiment 1 showed that tonal strings with shorter ISI were liked more than those with longer ISI while such difference was not found between symmetric and asymmetric strings without training. Additionally, Experiment 2 found both main effects of symmetry and ISI as well as an interaction. In particular, only asymmetric strings were more likely to be judged as grammatical when they were presented at a shorter ISI. Taken together, participants were sensitive to the fluency induced by the manipulation of ISI and sensitive to symmetry only after training. In sum, we conclude that objective speed influenced grammaticality judgments, implicit learning of tonal symmetry resulted in enhanced fluency, and that fluency may serve as a basis for grammaticality judgments.

## Introduction

Processing fluency refers to a personal feeling of ease when the individual is performing a cognitive operation (Reber et al., 2004b). As fluency is not the cognitive process one is performing, but rather the metacognitive experience of ease related to performing the process (Oppenheimer, 2008), it can be generated across a wide array of cognitive forms and thus has an influence on people’s various judgments. For example, fluent propositions or statements tend to be judged truer than disfluent ones (Begg et al., 1992; Reber and Schwarz, 1999; Hansen et al., 2008). The more fluently an item is processed, the more frequently it is judged as familiar (Whittlesea, 1993; Topolinski and Strack, 2009, 2010; Topolinski, 2012; Susser and Mulligan, 2015). Easily retrieved or processed stimuli are rated more likable (Zajonc, 1968; Bornstein and D’agostino, 1992; Reber et al., 1998; Reber et al., 2004a). The relationship between ease-of-processing and liking is an important issue in the theory of processing fluency (Reber et al., 2004a). The theory suggests that fluency signals a positive state of affairs, absence of threat and safety, which are characterized by hedonic marking (Winkielman and Cacioppo, 2001; Winkielman et al., 2003). This positivity is then interpreted as a positive reaction toward the stimulus, resulting in liking. Various studies have shown that higher ease-of-processing is associated with higher liking judgments (for a review, see Alter and Oppenheimer, 2009). Thus, the enhancement of preference may serve as an indicator to elicit processing ease in the task during which a stimulus is perceptually clarified. In summary, we speculated that the faster a stimulus was presented, the more fluently and pleasantly participants might felt.

Similarly, in the research field of implicit learning, letter strings clarifying more quickly can under some conditions be more likely to be classified as grammatical (Kinder et al., 2003). In the study of implicit learning, the artificial grammar learning (AGL) is a typical paradigm employed. In the AGL task, grammaticality judgment is a common procedure to evaluate whether implicit knowledge has been acquired. In particular, participants are instructed to classify if novel strings are grammatical or not after being trained on other grammatical strings (Reber, 1967). Fluency can play a role in AGL by affecting participants’ classification performance on grammatical strings. For instance, Kinder et al. (2003) found that faster clarification of test strings, leading to an increased feeling of fluency, increased the proportion of “grammatical” responses to test strings. Consistently, Scott and Dienes (2010) also manipulated the visual clarification speed and replicated the results that faster- clarifying strings were more often endorsed as grammatical, indicating that fluency indeed had an influence on the grammatical judgments in AGL task for briefly exposed test strings. In general, fluency may serve as a basis for the classification of grammatical strings at least in the visual modality, and when there are no other strong cues (Johansson, 2009).

With respect to the auditory modality, it presents a different sort of computational problem as stimuli in audition need to be processed over time rather the space of the visual modality (Tyler, 2003). Ling et al. (2016) adopted tonal symmetry (i.e., Chinese tonal strings constructed with symmetric structure) as materials and illustrated that the implicit acquisition of the tonal symmetry could reliably elicit processing fluency of symmetric strings. In particular, participants listened to symmetric training strings, and then, in the test phase, participants responded to each syllable of a string according to its tone (1/2/3/4) under the guise of a choice RT task. Results showed that participants responded faster toward tones in the second half of tonal symmetric strings than the asymmetric ones. Two questions arise from the findings. First, the RT effects found by Ling et al. (2016) only shows a correlation of fluency with test string grammaticality. More direct evidence for fluency playing a role in judgments would be provided by experimentally manipulating fluency directly. Second, Ling et al. (2016) presented all participants with symmetric strings in a training phase. This means a crucial question is left open: Was the training needed to lead people to implicitly learn to process the symmetry fluently, or rather do people have a pre-existing tendency to processing such symmetries fluently? Answering this question is important both for understanding the computational nature of implicit learning and also for understanding how we process symmetries more generally.

Based on previous studies, the current study aimed to examine how symmetry-based fluency might influence grammaticality judgments of tonal strings and to compare such an influence on the effect of fluency being experienced by speeding up the perception process of stimulus. The latter manipulation directly affects the time of processing of the whole stimulus, and thus can test whether people are sensitive to objective fluency, the claimed variable training is said to affect. While felt fluency may be the important psychological variable in determining liking (Forster et al., 2013), felt fluency is meant to track objective fluency.

## Overview of the Current Research

**FIGURE 1:**
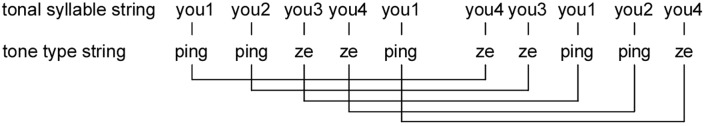
An example of tonal symmetry. The symmetry relation employed by Jiang et al. (2012) and Ling et al. (2016; and in the present study) is based on the use of Chinese tones in Tang dynasty poetry, in which the tones of ping (i.e., tone 1 and tone 2) regularly correspond to the tones of ze (i.e., tone 3 and tone 4). The symmetry relation was constructed according to a mirror inversion of ping and ze tones with the first five tones predicting the last five ones. e.g., if the tone type of the first syllable was ping, then the tone type of the sixth syllable was ze, and so on.

Following Jiang et al. (2012), in the current study, we defined the symmetry over the four tones (1–4) of Chinese syllables, which represent different phonetic characteristics in pitch. In Chinese, these four tones are categorized into ping tones (tone 1 and tone 2) and ze tones (tone 3 and tone 4). Similar to Jiang et al. (2012) participants were presented with sequences of 10 syllables where the tone types of the first five syllables of a sequence corresponded to those of the last five, e.g., if the first syllable was of ping tone, then the sixth syllable should be of ze tone, and a ping tone on the second syllable predicted a ze tone on the seventh syllable, and so on (**Figure 1**). In the training phase, participants were required to repeat back sequences constructed with this tonal symmetry; in the test phase, they were asked to respond to novel strings either following or violating the tonal symmetry. We controlled both chunks and repetition patterns at the level of syllables, tones and tone types (ping/ze).

Experiment 1 explored whether in such paradigm we could experimentally induce fluency by speeding up the presentation of tonal strings, analogous to the manipulation of clarifying speed on visual stimulus in previous studies (Kinder et al., 2003; Scott and Dienes, 2010). Specifically, the inter-stimulus interval (ISI) between each syllable of a string was manipulated. To further examine whether the tonal symmetry can directly enhance fluency without training, we carried out Experiment 1 with only a testing phase. Experiment 2 was same to Experiment 1 with two exceptions: firstly, there was a training phase on tonal symmetry before participants made responses to tonal strings; secondly, during the testing phase participants were instructed to make grammatical judgments instead of liking ratings. Being informed that the strings in training phase were constructed by a complex rule (i.e., the symmetry relation, which participants were not informed about), participants were required to classify whether the new strings in test phase were grammatical or not.

## Experiment 1

In Experiment 1, we orthogonally manipulated the symmetry and ISI. After each sequence, participants rated how much they liked the string. Scott and Dienes (2010) found that in visual cases, the faster a sequence was presented during a perceptual clarification task, the more fluent participants would felt. Thus for an auditory sequence, the faster presentation may lead to an increasing feeling of fluency and hence, to increasing liking, for the auditory stimuli.

Although the tonal symmetry indeed produced fluency after implicit acquisition (Ling et al., 2016), it remains unclear whether the symmetry can enhance fluency without a training phase. In order to clarify this issue, we instructed participants to make liking ratings without any training on symmetry.

We expected that the strings presented faster with short ISI, producing greater objective speed or fluency of processing should enhance preference ratings if fluency is indeed the relevant psychological variable determining response to the strings. The primary aim of the experiment was to determine that objective fluency affects liking of tonal strings. Finally, whether symmetric strings are liked more than asymmetric ones depends on whether tonal symmetry elicits fluency without training.

### Materials and Methods

#### Participants

Eighteen volunteers (8 female, aged 22–29, *M* = 25.22, *SD* = 1.96) from the university were recruited as participants in return for 30RMB. All participants were native Chinese speakers and none of them had hearing difficulties. The study was given approval by the Ethics Committee of the East China Normal University and written informed consent was obtained from each participant before the experiment.

#### Design

Experiment 1 used a 2 × 3 design: Symmetry (symmetric vs. asymmetric) by ISI (80 ms vs. 100 ms vs. 120 ms) were the within-subjects factors. The dependent variable was participants’ liking rating scores.

#### Materials

The tonal symmetry employed in the current study was comparable to that used by Jiang et al. (2012), though the precise materials were different. In this experiment, four tonal syllables (i.e., you1, you2, you3, and you4) were selected and categorized into two kinds according to Chinese tone types: “ping” (you1 and you2) and “ze” (you3 and you4). Each string comprised 10 tonal syllables and the first five tonal syllables’ tone types (pings or zes) predicted the tone types of the last five syllables as shown in **Figure 1**. That is, ping in the first half corresponds to ze in the second half in the same position in the tonal strings, and ze corresponds to ping in the same position.

The difference in materials between Experiments 1 and 2 was that there was no training phase in Experiment 1; the test strings used in Experiments 1 and 2 were identical despite different tasks. Here the materials of both experiments are introduced.

Based on the symmetry relation, we generated thirty-two symmetric tone type strings, of which 16 strings were chosen for the training phase for experiment 2 (see Supplementary Table S1), while the remaining 16 symmetric strings, combining with 16 asymmetric strings were used in the test phase for Experiments 1 and 2 (see Supplementary Table S2). Specifically, the 16 asymmetric test strings were created according to the 16 symmetric test strings by violating the symmetry relation in any two positions (as was done in Jiang et al., 2012, e.g., the asymmetric tone type string of “ping ping ze ping ze—ping ze ping ze ze” is generated from the symmetric tone type string of “ping ping ze ping ping—ze ze ping ze ze” by exchange the position of the fifth and sixth tone, resulting in violation of symmetry in these two positions). Each training tone type string was used to generate three different tonal syllable strings (e.g., the tone type string of “ping ping ze ze ping—ze ze ping ping ze” can generate three tonal syllable strings as follows: “you1 you2 you3 you4 you1—you4 you3 you1 you2 you4”; “you2 you1 you4 you3 you2—you3 you4 you2 you1 you3”; “you1 you2 you4 you3 you2—you4 you3 you2 you1 you3”). Thus for Experiment 2, 48 training tonal syllable strings were created in total (see Supplementary Table S3). Similarly, 32 test tonal syllable strings (16 symmetric strings and 16 asymmetric strings) were generated, one for each tone type string for both Experiments 1 and 2 (see Supplementary Table S4). All of the strings had no clear semantic interpretation. Each subject received the same training strings and test strings in the study.

Both repetition structure and chunking of the materials were controlled. The repetition structures of test strings, represented by a sequence of tone types, were different from any of the training strings. For example, the repetition structure of “ze ze ping ze ze” is “11211,” or that of “ping ping ze ze ping” is “11221” (cf. Vokey and Brooks, 1992). For the test strings, both symmetric and asymmetric ones had no same repetition structures, in terms of tones 1–4, as any of the training strings. Furthermore, we counterbalanced global associative chunk strength (GACS), anchor associative chunk strength (AACS) and mean feature frequency (MFF) between symmetric and asymmetric test tone type strings (**Table 1**). GACS of each test tone type string was the average frequency scores across all of the chunks (bigrams and trigrams) in the string and AACS was the frequency with which tone type chunks appeared in the first and last positions (e.g., Knowlton and Squire, 1994; Kuhn and Dienes, 2005; Jiang et al., 2012). MFF was calculated by averaging the number of times each tone type occurred in the training phase in each of all positions. Further, the chunks of symmetric and asymmetric strings were balanced along the same dimensions of tone types, as just described, and also in terms of tones 1–4 (see **Table 1**).

**Table 1:** Mean MFF and ACS for symmetric and asymmetric strings in terms of tone types (ping and ze), tones 1–4 (*M* ±*SD*).

	Tone types		Tones 1–4	
	Symmetric	Asymmetric	Symmetric	Asymmetric
MFF	720.00 ± 0.00	720.00 ± 0.00	359.96 ± 0.31	359.93 ± 0.30
Global ACS	233.72 ± 2.31	234.28 ± 1.39	51.56 ± 3.36	51.05 ± 3.46
ACS				
Anchor	52.88 ± 3.07	52.31 ± 3.23	10.69 ± 2.11	10.73 ± 1.89
ACS				

*MFF, mean feature frequency; ACS, associative chunk strength*.

We generated four tonal syllables by Chinese pronunciation software (*Xunfei* InterPhonic 2.30) with a female voice, each lasting for 300 ms. For each tonal syllable string, there was an interposed 600 ms interval between the fifth and sixth tonal syllables to produce a perceptual gap between the first half and second half of the string (cf. Mueller et al., 2010; Jiang et al., 2012). In addition, the ISI defined the interval of each tonal syllable and there were three types of ISI: 80 ms (short ISI), 100 ms (standard ISI), and 120 ms (long ISI). Thirty two tonal syllable strings were presented for each type of ISI (80 ms, 100 ms, and 120 ms, respectively), resulting in 96 syllable strings in all. In order to avoid participants noticing the difference between syllable strings with short and long ISIs, we restricted the pseudo-random order of the test sequences so as to prevent tonal syllable strings with short and long ISIs from being adjacent (cf. Buchner et al., 1997). The test strings were pseudo-randomly ordered once and that order presented to each subject.

#### Procedure

Participants were told that they were participating in a task studying subjective preference of different audio strings. They were required to listen to 96 strings in total, which consisted of 32 tonal syllable strings presented with each of three ISIs. In each trial, participants were asked to listen to a syllable string and rate how much they like it on a 9-point scale (1 = do not like it, 5 = indifferent, 9 = like it a lot) and we encouraged them to make use of the full range of the scale.

#### Data Analysis

Using Bayes factors, *B*, one degree of freedom effects are tested to assess strength of evidence (e.g., Wagenmakers et al., 2015; see Dienes, 2015; Sand and Nilsson, 2016, for the relevance of Bayes factors for implicit cognition); we also reported *p*-values so that readers can assess significance additionally. If the value of a *B* is above three, it provides evidence for the alternative hypothesis is substantial instead of the null hypothesis (Jeffreys, 1939). And a *B* lower than 1/3 indicates substantial evidence for the null instead of the alternative hypothesis. “Substantial” has the meaning of being just worth taking note of. Therefore, a *B* between 1/3 and 3 reveals that the data is insensitive for differentiating the alternative and null hypotheses (see Dienes, 2014). These conventions are guidelines and not absolute thresholds. *B*_H(0,x)_ is a Bayes factor employed to test the alternative hypothesis, represented as a half-normal with an *SD* of x, against *H*_0_, the null hypothesis [the “H” in *B*_H(0,x)_ represents half-normal]. Following Dienes (2014), we used a roughly expected effect size as the *SD* of a half-normal. Vitz (1964) found that increasing the speed of presentation of tonal stimuli enhanced liking by about 1 unit (on a 9-point scale) for a 50% increase in speed. We will take this estimate as a rough scale of effect that could be expected, and hence, in modeling our H1, set the SD of the half-normal to 1 Likert unit. For simplicity, we use the same model of H1 for all tests. Both *p*-values and *B*’s are reported; we will interpret all effects with respect to the *B*’s. As it happened, in all but one case a result significant at the 5% level corresponded to a *B* > 3 with the model of H1 we used (cf. Jeffreys, 1939, p. 359, for this rough but not guaranteed correspondence between *B* and *p*; if the obtained effect is roughly the size expected on a half-Normal model of H1 the correspondence typically obtains, Dienes, 2014).

### Results

**Table 2** shows the liking ratings for symmetric and asymmetric syllable strings presented at ISI of 80, 100, and 120 ms respectively.

**Table 2:** Mean liking ratings for symmetric and asymmetric strings presented at each type of ISI (*M* ±*SD*).

ISI	Liking ratings	
	Symmetric	Asymmetric
80 ms	5.52 ± 0.75	5.28 ± 0.59
100 ms	5.20 ± 0.62	5.23 ± 0.70
120 ms	5.06 ± 0.72	5.02 ± 0.64

**FIGURE 2:**
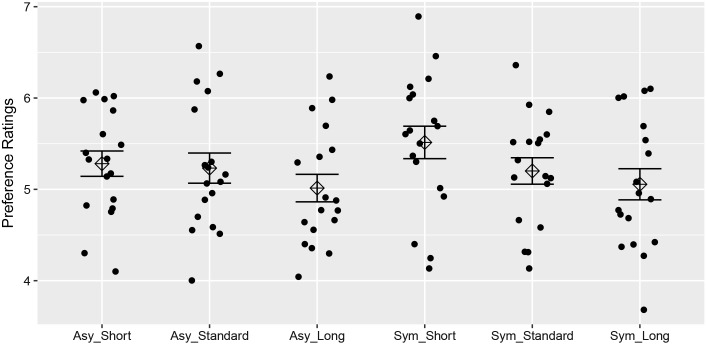
The preference ratings of symmetric and asymmetric strings under different ISI conditions. The response of each individual as well as the average response was illustrated with standard error bars.

The liking ratings were submitted to a 2 (symmetric vs. asymmetric) × 3 (80 ms vs. 100 ms vs.120 ms) within-subjects ANOVA. The analysis yielded a significant main effect of ISI, *F*(2,34) = 11.82, *p* < 0.001, $\eta_{p}^{2}$ = 0.41. Paired-samples *t*-tests (with sequential Bonferroni correction for significance testing) indicated evidence for the difference of liking ratings between short ISI (i.e., 80 ms) and long ISI (i.e., 120 ms), *t*(17) = 4.40, *p* < 0.001, *d* = 1.04, *B*_H(0,1)_ = 783.93, as well as for the difference between standard ISI (i.e., 100 ms) and long ISI (i.e., 120 ms), *t*(17) = 2.71, *p* < 0.05, *d* = 0.64, *B*_H(0,1)_ = 3.51. Although people gave higher liking ratings to the syllable strings when they were presented at ISI of 80ms than presented at ISI of 100 ms, the evidence was insensitive, *t*(17) = 2.50, *p* < 0.05, *d* = 0.59, *B*_H(0,1)_ = 2.31. In addition, there was no difference on liking ratings between symmetric strings and asymmetric ones, *F*(1,17) = 0.80, *p* = 0.38, $\eta_{p}^{2}$ = 0.05, *B*_H(0,1)_ = 0.22 (see **Figure 2**). There was no significant interaction between ISI and symmetry, *F*(2,34) = 1.00, *p* = 0.38, $\eta_{p}^{2}$ = 0.06. Furthermore, we divided the test strings into two halves. Paired-samples *t*-tests showed that symmetric strings were no more liked than asymmetric ones in the first half, *t*(17) = 0.19, *p* = 0.86, *d* = 0.04, *B*_H(0,1)_ = 0.22. Whereas in the second half, the evidence was insensitive, *t*(17) = 1.71, *p* = 0.11, *d* = 0.40, *B*_H(0,1)_ = 0.84, indicating that participants might have learned something about the symmetrical structure of the strings during the test of Experiment 1.

### Discussion

In Experiment 1, tonal strings with shorter ISI were rated more likable than those with longer ISI, which indicated the validity of the fluency manipulation in auditory modality according to the claims of a link between fluency and liking (Reber et al., 1998; Winkielman and Cacioppo, 2001; Winkielman et al., 2003). There was evidence that symmetric strings were no more liked than the asymmetric ones in the absence of training phase. In sum, we show that objective fluency can determine liking of tonal strings. Further, the symmetry we use is difficult to process fluently without training.

## Experiment 2

Experiment 2 used the same manipulation together with training the symmetry relation. To further investigate the role of the fluency induced by tonal symmetry in AGL, participants were instructed to classify whether the novel strings in test phase were grammatical or not. Jiang et al. (2012) revealed that being trained with symmetric strings, people could implicitly learn the rule of tonal symmetry and classify above chance in a later grammatical judgment task. Ling et al. (2016) further revealed that after implicit learning the symmetric rule, fluency in the form of reaction times was enhanced for tonal symmetric strings compared to asymmetric strings. However, we have not yet shown that objective fluency leads to an enhanced rate of grammaticality judgments; this step is needed for showing that implicit learning of tonal symmetry works via creating feelings of fluency. Hence, we employed the same manipulation of ISIs in Experiment 2 to further explore how acquired fluency elicited by symmetry and fluency enhanced by shorter ISIs may affect grammatical judgments.

For measuring the implicit nature of any knowledge of the tonal symmetry, we used the structural knowledge attributions of Dienes and Scott (2005). Knowledge of the structure of stimuli is unconscious according to higher-order theories of consciousness (e.g., Lau and Rosenthal, 2011) when people are not aware of knowing the structural properties they actually use to classify. That is, being conscious or unconscious is a matter of making a metacognitive judgment about one’s knowledge. Structural knowledge could be unconscious when people either did not know they had known at all, i.e., they thought they were purely guessing, or else if they were aware they had knowledge but they had no idea what that knowledge was, i.e., if they used feelings of intuition or familiarity, but had no idea what the basis of those feelings was. Conversely, structural knowledge is clearly conscious if people could state the rules or recollections they used to classify a test stimulus. Thus, after each classification decision, participants were required to state the basis of that classification, whether it was based on random responding, feelings of intuition or familiarity, or rules or recollections (for previous use of these measures see also e.g., Hamrick and Rebuschat, 2012; Kiyokawa et al., 2012; Neil and Higham, 2012; Norman and Price, 2012; Williams and Rebuschat, 2012; Kemény and Lukács, 2013; Li et al., 2013; Rebuschat et al., 2013).

### Materials and Methods

#### Participants

Twenty-four universities students (13 female, aged 19–36, *M* = 23.50, *SD* = 3.79) participated in the experiment in return for 30RMB with their written informed contents obtained before the experiment. All participants speaking Chinese as their mother language and no one had a history of hearing deficiency. Ethical approval was attained from Ethics Committee of the East China Normal University.

#### Design

The independent variables were ISI with three levels (80, 100, 120 ms) and symmetry status (symmetric vs. asymmetric). The dependent variables were the accuracy and the endorsement rate of grammatical judgments.

#### Materials

Forty-eight symmetric strings generated from the tonal symmetry were used as training strings (see Materials and Methods of Experiment 1), each with an ISI of 100 ms. The test strings were identical to those used in Experiment 1.

#### Procedure

During the training period, participants were exposed to 144 symmetric tonal syllable strings in sum, consisting of three blocks of 48 strings presented randomly (i.e., the presentation order randomized anew for each subject) in each block. A 500 ms warning tone was presented at the beginning of each trial, followed by a 4000 ms tonal string and a 5000 ms blank. Participants listened to each string carefully and silently repeated it during the blank before the next trial. This phase lasted about 27 min.

After training, we told participants that the strings they heard previously were created by a specific rule. And then, a set of 96 new strings were presented pseudo-randomly and participants were asked to classify whether each given string was grammatical. After each classification, we asked participants to indicate the basis of their decision (choosing one option from “guess,” “intuition,” “familiarity,” “memory” and “rules”). Specifically, “Guess” suggested that the classification was based on nothing, it could as well be based on a coin toss; “intuition” suggested that the classification was based on a feeling that could not be explained further, i.e., participants were confident in their decision but had no idea why it was right; “familiarity” suggested that the classification was based on how familiar or unfamiliar the string felt but could not explicate the reason; “memory” suggested that the classification was based on a recollection they had or failed to have; “Rules” suggested the classification was based on a rule that participants could state if asked (e.g., Fu et al., 2010; Chen et al., 2011).

### Results

As before results are interpreted with respect to Bayes factors. The dependent variable in Experiment 2 was the proportion of grammaticality judgments. Jiang et al. (2012) found a 14% difference in endorsements between grammatical and non-grammatical items for their similar paradigm; or 7% in terms of average correct above chance. Thus, we will use these figures as the SD for a half-normal in modeling H1.

#### Learning and Unconscious Knowledge

The proportion of correct response was calculated by 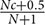, (*Nc* is the number of correct responses, and *N* is the total number of responses), this correction is useful if participants have low N under some conditions (similar to Laplace’s correction to a proportion, but using a “unit information prior,” i.e., a prior worth one observation; cf. Baguley, 2012, pp. 83 and 397).

The overall classification performance was 0.56, (*SD* = 0.09), better than chance (50%), *t*(23) = 3.00, *p* < 0.01, *d* = 0.61, *B*_H(0,7%)_ = 26.39, indicating that learning occurred. Guess, intuition and familiarity were combined as implicit attributions, indicating that the structural knowledge was unconscious. While memory and rule were considered as explicit attributions, revealing that the structural knowledge was conscious (see e.g., Wan et al., 2008; Mealor and Dienes, 2012). We excluded explicit attributions in further analyses since the percentage of them was less than 4%. The overall classification performance of implicit attributions was 0.56 (*SD* = 0.09), *t*(23) = 3.16, *p* < 0.01, *d* = 0.64, *B*_H(0,7%)_ = 39.69, indicating that there was implicit knowledge.

#### The Fluency Effects Arise from ISIs and Symmetry

A 2 × 3 factorial analysis of variance on endorsement rates yielded a main effect of symmetry, *F*(1,23) = 8.99, *p* = 0.01, $\eta_{p}^{2}$ = 0.28, *B*_H(0,14%)_ = 25.96, and a main effect of ISI, *F*(2,46) = 11.29, *p* < 0.001, $\eta_{p}^{2}$ = 0.33, which were qualified by a significant interaction between symmetry and ISI, *F*(2,46) = 4.90, *p* = 0.01, $\eta_{p}^{2}$ = 0.18.

**FIGURE 3:**
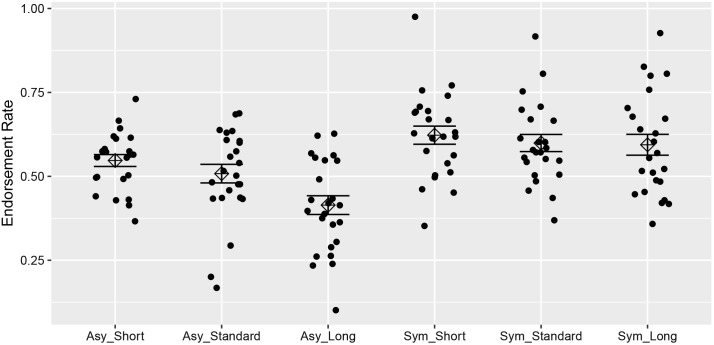
The endorsement rate of symmetric and asymmetric strings under different ISI conditions. The response of each individual as well as the average response was illustrated with standard error bars.

To unpack the interaction, paired-samples *t*-tests with sequential Bonferroni correction (Hochberg, 1988) were undertaken looking at the difference between endorsement rates at each ISI for grammatical and ungrammatical strings respectively (Bonferroni correction obviates the need for omnibus simple effect F’s; e.g., Lewis, 1993). For asymmetric strings, there was a difference between endorsement rate at the standard and long ISI, *t*(23) = 3.89, *p* = 0.001, *d* = 0.79, *B*_H(0,14%)_ = 320.03, and between the short and long ISI, *t*(23) = 6.76, *p* < 0.001, *d* = 1.38, *B*_H(0,14%)_ = 29.66 × 10^7^. However, the evidence was insensitive for the difference between endorsement rate at the short and standard ISI, *t*(23) = 1.79, *p* > 0.05, *d* = 0.36, *B*_H(0,14%)_ = 1.30. For symmetric strings, there was evidence for no difference between endorsement rate at the standard ISI and long ISI, *t*(23) = 0.18, *p* > 0.05, *d* = 0.04, *B*_H(0,14%)_ = 0.25. For that at the short and standard ISI, there was no evidence one way or the other, *t*(23) = 0.88, *p* > 0.025, *d* = 0.18, *B*_H(0,14%)_ = 0.44, and likewise no evidence for a difference between the short and long ISI, *t*(23) = 1.1, *p* > 0.0175, *d* = 0.22, *B*_H(0,14%)_ = 0.57 (see **Figure 3**). For symmetric strings, the most sensitive test should be that between the long and short ISI. That is, while the effect of ISI was decreased for symmetric rather than asymmetric strings, we cannot conclude that it was eliminated.

### Discussion

In Experiment 2, we found that the symmetric strings were more often endorsed as grammatical than the asymmetric ones, which was consistent with Jiang et al. (2012) and Li et al. (2013). Further, people’s endorsements were also sensitive to manipulated fluency, i.e., the speed with which the display of each string was completed, replicating Kinder et al. (2003) with an auditory rather than visual paradigm.

There was an interaction between symmetry and ISI, indicating that the fluency effect from ISI was stronger for asymmetric than symmetric strings. This may be because identifying symmetry does not rely on manipulated perceptual fluency and perceptual fluency is only used in the absence of other identifying information (Whittlesea and Leboe, 2000; Johansson, 2009; Scott and Dienes, 2010). Alternatively, it may be because the fluency from each source combines non-additively. Given that Ling et al. (2016) found that symmetry produced fluency, and the current experiment shows that fluency promotes endorsements of grammaticality, the simplest most coherent explanation is that the fluency induced by ISI and symmetry combine non-additively.

## General Discussion

The present research aimed to illuminate whether fluency elicited by the implicit acquisition of tonal symmetry is the basis of judgments of liking or well-formedness of symmetrical strings. Considering the close relationship between processing fluency and liking (Winkielman and Cacioppo, 2001; Reber et al., 2004a; Alter and Oppenheimer, 2009), we chose preference rating as a measurement of fluency. In Experiment 1, without a training phase, participants listened to the tonal strings and rated how much they liked each string. Symmetry status had no influence on liking ratings, indicating that tonal symmetry does not produce fluency or at least fluency that is used to determine affective response, without implicit acquisition of the symmetry. In addition, the strings presented with shorter ISI were rated more likable, revealing that participants did use fluency to inform subjectively. We conclude that the symmetry in the test strings did not produce fluency without a training phase.

Experiment 2 investigated the role of fluency produced by string presentation rate on grammaticality judgments. In detail, the strings presented faster with shorter ISI were more likely to be endorsed as grammatical than the strings presented with a longer ISI. This main effect revealed that the classification of the tonal strings was based on the general feeling of processing ease. In other words, the strategy participants used in grammatical judgments was a fluency heuristic, consistent with the conception put forward by Kinder et al. (2003) in a letter-based AGL task. Further, symmetric strings were endorsed more than asymmetric strings, indicating an influence of tonal symmetry on the grammatical judgments after being implicitly learned.

Notably, an interaction was found between ISI and symmetry. In particular, asymmetric strings were more likely to be judged as grammatical when they were presented at a shorter ISI while the endorsement rate of symmetric strings was less influenced by the manipulation of ISI. This interaction, indicating that the fluency effect from ISI was stronger for asymmetric than symmetric strings, can be explained by the “discrepancy-attribution hypothesis,” which was first put forward by Whittlesea and Williams (1998). This hypothesis proposes that a feeling of familiarity occurs when perceiving a discrepancy between the actual and expected fluency of processing (Whittlesea and Williams, 1998, 2000, 2001). Participants unconsciously attributed the perceived discrepancy to a prior experience of the stimulus, leading to a higher rate of false alarm for asymmetric strings in the current study. Unlike the findings of Milhau et al. (2017), which demonstrated that positive stimuli enhanced motor fluency and facilitated the realization of fluent lateral movements, the fluency induced from ISI and symmetry in the present research combined non-additively. This might because the fluency produced by the implicit acquisition of the symmetry may have been sufficient for facilitating grammaticality judgments. For asymmetric strings, the lower starting level of fluency may have allowed the pure effect of manipulated perceptual fluency to occur.

Computationally, detecting and using spatial symmetry to speed processing may be achieved in a spatial way in terms of neural coding (Tyler, 2003); however, auditory symmetries are not extended in space but time, and this in principle may require different computational mechanisms. Thus, it is not obvious that auditory symmetries would speed processing. Further, while learning any regularity could lead to fluency, processing regularities need not speed processing in order to be detected as regularities (Scott and Dienes, 2010); and if the regularity does create fluency, fluency need not be used to inform judgments (Johansson, 2009). Thus, the current demonstration that learned auditory symmetries produce fluency that people use to classify is not trivial. Indeed, the finding raises the key theoretical question of how the symmetries could be learned. Symmetry constitutes a supra-finite state type of structure, and thus requires a memory buffer (Li et al., 2013). Rodriguez et al. (1999) showed how the Simple Recurrent Network (SRN), a connectionist network with a learnable memory buffer, could in at least a simple case become a graded supra-finite state learning device. As the SRN is one of the more promising models of implicit learning in general (Cleeremans and Dienes, 2008), it may provide a model of implicit learning of symmetries.

## Ethics Statement

The current study was implemented in conformity to the recommendations of the Ethical Committee of East China Normal University. Informed consent of all participants was obtained in line with the Declaration of Helsinki. The protocol was approved by the Ethical Committee of East China Normal University.

## Author Contributions

FQ, FL, and XL designed and implemented the study. FQ, FS, and XL analyzed the data. XG, LL, and LZ interpreted the data. FQ, FS, and ZD wrote the manuscript.

## Conflict of Interest Statement

The authors declare that the research was conducted in the absence of any commercial or financial relationships that could be construed as a potential conflict of interest.
