# Supplementary material for: Tonal Symmetry Induces Fluency and Sense of Well-Formedness
Source: Front Psychol. 2018 Feb 19;9:165. doi: 10.3389/fpsyg.2018.00165 (PMC5829615; doi:10.3389/fpsyg.2018.00165)
Supplement: Supplementary file 1 [file Table_1.docx]

Supplementary Material

**Tonal symmetry induces fluency and sense of well-formedness**

**Fuqiang Qiao**^†^**, Fenfen Sun**^†^**, Fengying Li, Xiaoli Ling, Li Zheng, Lin Li, Xiuyan Guo*, Zoltan Dienes**

*** Correspondence:** Xiuyan Guo: xyguo@psy.ecnu.edu.cn; wlkc_xyguo@126.com

# Supplementary Tables

| **Table S1** *Tone type strings used in training phase for Experiment 2* | |
| --- | --- |
| **Tone type strings** | **Tone type strings** |
| \| 1 ping ping ping ping ping ze ze ze ze ze \|  \| \| --- \| --- \| \| 2 ping ping ping ze ping ze ze ze ping ze \|  \| \| 3 ping ping ze ze ze ze ze ping ping ping \|  \| \| 4 ping ping ze ping ze ze ze ping ze ping \|  \| \| 5 ping ze ping ping ping ze ping ze ze ze \|  \| \| 6 ping ze ping ping ze ze ping ze ze ping \|  \| \| 7 ping ze ze ping ze ze ping ping ze ping \|  \| \| 8 ping ze ze ping ping ze ping ping ze ze \|  \| | \| 9 ze ping ping ze ping ping ze ze ping ze \| \| --- \| \| 10 ze ping ping ze ze ping ze ze ping ping \| \| 11 ze ping ze ze ze ping ze ping ping ping \| \| 12 ze ping ze ze ping ping ze ping ping ze \| \| 13 ze ze ping ping ping ping ping ze ze ze \| \| 14 ze ze ping ze ping ping ping ze ping ze \| \| 15 ze ze ze ze ze ping ping ping ping ping \| \| 16 ze ze ze ping ze ping ping ping ze ping \| |

| **Table S2** *Tone type strings used in test phase for Experiment 1 & Experiment 2* | |
| --- | --- |
| **Symmetric strings** | **Asymmetric strings** |
| \| 1 ping ping ping ze ze ze ze ze ping ping \| \| --- \| \| 2 ping ping ping ping ze ze ze ze ze ping \| \| 3 ping ping ze ze ping ze ze ping ping ze \| \| 4 ping ping ze ping ping ze ze ping ze ze \| \| 5 ping ze ping ze ping ze ping ze ping ze \| \| 6 ping ze ping ze ze ze ping ze ping ping \| \| 7 ping ze ze ze ze ze ping ping ping ping \| \| 8 ping ze ze ze ping ze ping ping ping ze \| \| 9 ze ping ping ping ping ping ze ze ze ze \| \| 10 ze ping ping ping ze ping ze ze ze ping \| \| 11 ze ping ze ping ze ping ze ping ze ping \| \| 12 ze ping ze ping ping ping ze ping ze ze \| \| 13 ze ze ping ze ze ping ping ze ping ping \| \| 14 ze ze ping ping ze ping ping ze ze ping \| \| 15 ze ze ze ze ping ping ping ping ping ze \| \| 16 ze ze ze ping ping ping ping ping ze ze \| | \| 1 ping ping ping ze ze ping ze ze ze ping \| \| --- \| \| 2 ping ping ze ze ze ping ze ze ping ping \| \| 3 ping ping ze ze ze ze ping ping ping ze \| \| 4 ping ping ze ping ze ping ze ping ze ze \| \| 5 ping ze ping ze ze ping ping ze ping ze \| \| 6 ping ze ping ze ping ze ze ze ping ping \| \| 7 ping ze ze ze ping ze ze ping ping ping \| \| 8 ping ze ze ze ze ping ping ping ping ze \| \| 9 ze ping ping ping ze ping ping ze ze ze \| \| 10 ze ping ping ping ping ze ze ze ze ping \| \| 11 ze ping ze ping ping ze ze ping ze ping \| \| 12 ze ping ze ping ze ping ping ping ze ze \| \| 13 ze ze ping ze ping ze ping ze ping ping \| \| 14 ze ze ping ping ping ping ze ze ze ping \| \| 15 ze ze ping ping ping ze ping ping ze ze \| \| 16 ze ze ze ping ping ze ping ping ping ze \| |

| **Table S3** *Tonal syllable strings used in training phase for Experiment 2* | |
| --- | --- |
| **Tonal syllable strings** | **Tonal syllable strings** |
| \| 1 you3you3you4you3you4you1you1you2you2you2 \| \| --- \| \| 2 you1you1you2you2you2you3you4you3you3you4 \| \| 3 you4you1you3you3you4you2you4you2you1you1 \| \| 4 you2you4you2you1you2you3you1you4you4you3 \| \| 5 you4you4you3you1you3you2you2you2you3you1 \| \| 6 you2you1you2you4you1you3you3you3you1you4 \| \| 7 you1you1you4you3you3you4you4you2you1you2 \| \| 8 you3you4you1you1you2you2you1you3you4you4 \| \| 9 you1you3you4you1you3you4you2you2you3you1 \| \| 10 you3you2you2you4you1you2you3you4you1you3 \| \| 11 you4you1you1you3you4you2you3you4you2you2 \| \| 12 you2you3you4you2you2you3you1you1you4you4 \| \| 13 you3you2you4you3you1you1you4you2you1you3 \| \| 14 you1you4you2you1you4you3you2you3you3you2 \| \| 15 you3you3you1you4you1you2you2you4you2you4 \| \| 16 you2you1you3you1you4you3you3you2you4you2 \| \| 17 you4you4you3you3you4you2you1you1you2you1 \| \| 18 you2you1you1you1you2you3you4you4you4you3 \| \| 19 you3you1you3you3you4you1you4you1you2you2 \| \| 20 you2you3you2you1you1you3you1you4you3you4 \| \| 21 you3you4you3you2you3you1you2you1you4you2 \| \| 22 you1you2you1you3you2you3you3you4you1you4 \| \| 23 you2you1you4you3you4you3you4you1you2you2 \| \| 24 you4you3you1you2you2you1you1you3you4you3 \| | \| 25 you2you4you4you1you4you3you1you2you3you2 \| \| --- \| \| 26 you4you1you1you4you1you2you3you3you2you4 \| \| 27 you3you1you2you3you4you2you4you3you1you2 \| \| 28 you2you3you4you1you1you4you2you1you3you4 \| \| 29 you4you1you4you3you2you1you3you2you2you4 \| \| 30 you1you4you2you2you3you3you2you3you4you1 \| \| 31 you3you3you1you4you2you1you1you4you2you3 \| \| 32 you2you1you3you2you3you4you4you1you4you2 \| \| 33 you4you4you3you4you3you1you2you2you1you1 \| \| 34 you1you2you2you1you2you3you4you3you4you4 \| \| 35 you4you1you4you3you3you1you4you2you2you2 \| \| 36 you1you3you1you2you2you3you2you3you4you4 \| \| 37 you3you4you3you1you4you2you2you1you3you1 \| \| 38 you1you1you1you3you2you4you4you3you2you3 \| \| 39 you2you1you3you3you4you4you3you1you2you1 \| \| 40 you3you4you2you1you2you1you1you3you3you4 \| \| 41 you1you3you3you2you4you3you2you1you4you1 \| \| 42 you4you2you2you4you1you1you3you4you1you3 \| \| 43 you3you2you1you4you4you1you3you4you2you2 \| \| 44 you1you4you4you2you2you4you1you2you3you3 \| \| 45 you4you2you4you4you1you1you3you2you1you3 \| \| 46 you2you4you1you1you4you3you1you3you4you2 \| \| 47 you4you3you1you4you1you2you2you3you1you4 \| \| 48 you2you2you3you1you4you4you3you2you3you1 \| |

| **Table S4** *Tonal syllable strings used in test phase for Experiment 1 & Experiment 2* | |
| --- | --- |
| **Symmetric strings** | **Asymmetric strings** |
| \| 1 you1you4you2you4you2you4you1you3you2you3 \| \| --- \| \| 2 you1you4you3you3you4you4you1you2you1you2 \| \| 3 you2you3you4you3you1you4you2you1you1you4 \| \| 4 you1you2you3you3you2you4you3you2you1you4 \| \| 5 you1you4you1you3you4you3you2you3you2you1 \| \| 6 you1you2you2you3you3you4you4you3you2you1 \| \| 7 you3you4you4you3you2you1you1you1you2you4 \| \| 8 you3you4you2you3you4you1you2you3you2you1 \| \| 9 you3you4you1you2you3you1you2you4you3you2 \| \| 10 you3you1you4you2you3you1you4you2you4you2 \| \| 11 you3you1you2you2you1you2you3you4you4you4 \| \| 12 you1you2you4you1you2you3you4you1you3you3 \| \| 13 you2you2you2you1you4you3you3you4you3you1 \| \| 14 you4you2you1you1you4you1you3you3you3you2 \| \| 15 you4you3you3you1you2you2you1you1you4you3 \| \| 16 you3you1you4you1you2you2you3you2you4you3 \| | \| 1 you1you2you2you4you4you1you3you3you4you2 \| \| --- \| \| 2 you4you3you2you4you2you3you1you4you2you1 \| \| 3 you1you4you3you4you2you3you3you1you2you1 \| \| 4 you1you2you3you4you3you2you3you4you2you1 \| \| 5 you2you4you1you4you3you1you2you4you2you3 \| \| 6 you1you1you4you3you4you3you2you2you2you3 \| \| 7 you1you3you4you3you4you1you2you2you2you4 \| \| 8 you1you3you1you3you2you4you4you3you2you1 \| \| 9 you4you1you2you2you4you1you2you3you3you3 \| \| 10 you4you4you1you2you2you2you3you3you4you1 \| \| 11 you1you2you4you2you3you1you3you1you4you4 \| \| 12 you4you2you4you2you1you3you3you1you4you2 \| \| 13 you4you3you3you2you1you4you1you1you2you3 \| \| 14 you3you4you1you1you2you4you1you2you3you3 \| \| 15 you3you2you4you1you4you2you1you1you3you4 \| \| 16 you4you1you2you1you2you3you4you3you3you2 \| |
